# CTNet: a convolutional transformer network for EEG-based motor imagery classification

**DOI:** 10.1038/s41598-024-71118-7

**Published:** 2024-08-30

**Authors:** Wei Zhao, Xiaolu Jiang, Baocan Zhang, Shixiao Xiao, Sujun Weng

**Affiliations:** Chengyi College, Jimei University, Xiamen, 361021 China

**Keywords:** Brain-computer interface (BCI), Motor imagery (MI), Transformer, Convolutional neural networks (CNN), Neural decoding, Rehabilitation, Computer science

## Abstract

Brain-computer interface (BCI) technology bridges the direct communication between the brain and machines, unlocking new possibilities for human interaction and rehabilitation. EEG-based motor imagery (MI) plays a pivotal role in BCI, enabling the translation of thought into actionable commands for interactive and assistive technologies. However, the constrained decoding performance of brain signals poses a limitation to the broader application and development of BCI systems. In this study, we introduce a convolutional Transformer network (CTNet) designed for EEG-based MI classification. Firstly, CTNet employs a convolutional module analogous to EEGNet, dedicated to extracting local and spatial features from EEG time series. Subsequently, it incorporates a Transformer encoder module, leveraging a multi-head attention mechanism to discern the global dependencies of EEG's high-level features. Finally, a straightforward classifier module comprising fully connected layers is followed to categorize EEG signals. In subject-specific evaluations, CTNet achieved remarkable decoding accuracies of 82.52% and 88.49% on the BCI IV-2a and IV-2b datasets, respectively. Furthermore, in the challenging cross-subject assessments, CTNet achieved recognition accuracies of 58.64% on the BCI IV-2a dataset and 76.27% on the BCI IV-2b dataset. In both subject-specific and cross-subject evaluations, CTNet holds a leading position when compared to some of the state-of-the-art methods. This underscores the exceptional efficacy of our approach and its potential to set a new benchmark in EEG decoding.

## Introduction

The brain-computer interface (BCI) systems are at the forefront of technological advancements, providing a novel avenue for users to communicate with external devices by transforming neural signals into real-time, executable commands^1–3^. Brain signals can be captured through several non-invasive methods, such as Electroencephalography (EEG), Magnetoencephalography (MEG), and Functional Magnetic Resonance Imaging (fMRI). Among these, EEG has been widely used for measuring electrical activity on the scalp, due to its superior temporal resolution, portability, and simplicity in operation^4^.

Motor imagery (MI), a prominent subject in BCI research, involves the mental simulation of a physical action without actual motor execution^5^. The integration of MI with EEG (MI-EEG) has seen notable success in the field of neurological rehabilitation^6–8^, in aiding individuals with disabilities^9–11^, and in non-medical applications such as gaming^12^, drone control^13^ and smart home^14^. A significant challenge in BCI technology is the accurate interpretation of human intentions from brain signals, which are often hampered by a low signal-to-noise ratio and non-stationary nature, further complicated by a range of noise sources such as biological artifacts, environmental interference, and electronic equipment. Consequently, constructing a robust MI-EEG BCI system capable of functioning effectively in diverse scenarios and autonomously extracting distinctive features from complex EEG signals becomes crucial.

In the past, researchers have employed machine learning techniques to devise a range of algorithms for classifying MI. These methods typically involve processing MI-EEG signals through three key stages: preprocessing, feature extraction, and classification. The common spatial pattern (CSP) method has been developed to perform intricate and crucial feature extraction^15^. It achieves this by identifying a weight vector that maximizes the variance difference between the projected EEG signals. Owing to its effectiveness, a variety of CSP-based algorithms have been designed, including filter bank CSP (FBCSP)^16^, discriminative filter band CSP (DFBCSP)^17^, and sparse filter band CSP (SFBCSP)^18^. In the classification stage, the features extracted from EEG trials are categorized into different MI tasks using various classifiers, such as linear discriminant analysis (LDA)^19,20^, random forest (RF)^21^, support vector machine (SVM)^18,22^, and extreme learning machine (ELM)^23^. However, the performance of these classifiers heavily depends on the hand-crafted extracted features, which are unstable and may lead to poor results^24^.

Recent rapid developments in deep learning (DL) have given rise to many remarkable end-to-end models, catalyzing major advances in computer vision and natural language processing^25,26^. These models stand out for their ability to independently extract intrinsic features from input data, eliminating the need for prior knowledge. This has inspired a wave of innovation in MI-EEG classification, where researchers have explored a diverse array of deep learning architectures, including convolutional neural networks (CNN)^27–32^, recurrent neural networks (RNN)^33–35^, temporal convolutional network (TCN)^36,37^, deep belief networks (DBN)^38^, auto-encoders(AE)^39^, and various Hybrid Models^40,41^, each contributing uniquely to the field.

CNN, a prevalent model in deep learning (DL), excels in extracting local and spatial patterns^42,43^. Several streamlined CNN architectures, such as ShallowConvNet^27^, DeepConvNet^27^, EEGNet^28^, and MCNN^29^, have been utilized to classify different MI-EEG tasks using raw signals, demonstrating impressive performance. However, CNNs, limited by their kernel size, often struggle to capture long-term dependencies critical in EEG time series analysis. On the other hand, RNN is a DL architecture tailored for time-series data, including EEG signals. Variants like long short-term memory (LSTM)^33,34^ and gated recurrent unit (GRU)^35^, which evolve from traditional RNN architecture, are employed to extract temporal features in classifying MI-EEG tasks. Yet, these models face limitations in parallel training and capturing long-term dependencies. Additionally, TCN represents a CNN variation specifically designed for time series modelling and classification^44^. Unlike standard CNNs, TCNs can exponentially increase their receptive field size with only a linear rise in parameter count^36,37^. While TCNs are adept at discerning temporal dependencies within sequences, they may fall short in effectively extracting complex features. Furthermore, Some studies have explored hybrid architectural models for MI-EEG classification, integrating the strengths of various approaches^40,41^.

Lately, the transformative success of the Transformer based on self-attention (SA) model^45^ in natural language processing and computer vision has sparked the development of several innovative models for MI-EEG classification. Within the Transformer architecture, the extensive receptive field of the self-attention block is utilized to grasp global information, leading to enhanced performance.

Transformer architecture was initially developed to tackle the complexities of machine translation within the field of Natural Language Processing (NLP), where grasping the context and interrelationships within extensive sequences is essential^45^. This architecture, refined for sophisticated sequence modeling in NLP, is well-suited for EEG signal processing. The inherent capabilities of Transformers to identify and highlight critical elements of a sequence, manage long-range dependencies, and process multiple data points concurrently provide a powerful framework for decoding the complex patterns prevalent in EEG signals. These attributes have enabled Transformers to be effectively applied across a range of EEG-related tasks. Such applications include MI classification^46–48^, seizure prediction^49^ and detection^50^, sleep stage classification^51^, and emotion recognition^52^, each benefiting from the model's ability to analyze and interpret intricate neural activities. Lately, several models utilize the self-attention mechanism of the Transformer, which provides an extensive receptive field to capture global information, thereby enhancing performance. However, these models often overlook the importance of learning local features, which are crucial for EEG signal decoding^46–48^. While the decoding accuracy of EEG signals using these Transformer-based approaches has seen improvements over previous methods, there remains considerable scope for further enhancement.

To address these challenges, we introduce a convolutional Transformer network, CTNet, a hybrid model that couples the strengths of both CNN and Transformer architectures. CTNet is an end-to-end network model that sequentially integrates a convolution module, a transformer encoder module, and a classifier. Initially, CTNet employs multi one-dimensional convolutional layers to extract local temporal and spatial features from EEG trials. Subsequently, it utilizes a Transformer encoder with multi-head attention (MHA) with the global receptive field to highlight the most significant features. Finally, simple fully-connected layers are employed to obtain the decoding results. We conducted thorough comparative experiments on the BCI IV-2a dataset, which includes a four-category MI-EEG classification, and the BCI IV-2b dataset, which comprises a two-category MI-EEG classification, demonstrating the outstanding performance of the CTNet model across different classification tasks. The principal contributions are outlined as follows:

1. We introduce a novel high-performance CTNet model, which effectively leverages CNN for local feature extraction and employs the Transformer encoder to learn global features from EEG trials.
2. Our proposed model establishes a new benchmark, with CTNet leading the field in both subject-specific and cross-subject evaluations compared to the current state-of-the-art methods on the BCI IV-2a and IV-2b datasets. This demonstrates CTNet's robust generalization capabilities and highlights its potential as a new standard for EEG decoding.
3. We undertake comprehensive experiments to thoroughly examine the impact of the Transformer encoder and the data augmentation, as well as the convolutional and attention parameters.
4. For the sake of reproducibility and further research, the code and trained models  have been released at https://github.com/snailpt/CTNet.

The remainder of this paper is structured as follows: Section “Related Work” provides an overview of pioneering research in the relevant field. Section “Materials and Methods” details the datasets used and describes our proposed algorithm. In Section “Experimental Results”, we present the performance results of our algorithm as obtained through experimental evaluation. Section “Discussion” delves into a discussion of the key findings of our study. Finally, Section “Conclusions” concludes the paper with our closing observations and insights.

## Related work

### Motor imagery classification with CNN

CNN has shown effectiveness in automatically extracting spatial features and classifying EEG signals, and it has gradually led to superior performance in MI-EEG data analysis tasks. Schirrmeister et al. introduced ShallowConvNet and DeepConvNet architectures inspired by the FBCSP transformation^27^. The ShallowConvNet comprises two convolutional layers, an average pooling layer, and a fully connected layer with a softmax classifier. The first convolution operates across time, while the second convolution functions across space, serving as an analogue to the CSP spatial filter. Compared to ShallowConvNet, DeepConvNet enhances its feature network by incorporating three additional convolutional-pooling blocks. Each of these blocks consists of a convolutional layer followed by a pooling layer, with all pooling operations in DeepConvNet, utilizing max pooling for optimal feature extraction. Lawhern et al. developed the notable EEGNet architecture, an advancement over the ConvNet structure^28^. EEGNet is composed of three types of convolution operations: temporal convolution, channel depth-wise convolution, and separable convolution. EEGNet stands out as a versatile architecture suitable for various BCI paradigms, achieving impressive results even when trained on limited data sets. Chowdhury et al. conducted an in-depth study on EEGNet and introduced a five-branch CNN architecture (EEGNet fusion V2) aimed at enhancing cross-subject motor imagery classification^31^. Each branch of this network incorporates EEGNet with varied hyperparameters to optimize feature fusion and classification performance. Their innovative approach demonstrated promising results across multiple datasets including eegmmidb, BCI IV-2a, and BCI IV-2b, significantly outperforming established models such as EEGNet, ShallowConvNet, and DeepConvNet in cross-subject scenarios. Ingolfsson introduced EEG-TCNet, a novel network that integrates EEGNet and TCN in a sequential manner^36^. This network is characterized by its low memory usage and reduced computational complexity, making it particularly well-suited for embedded classification in resource-constrained environments, such as edge devices. When evaluated on the BCI IV-2a dataset, EEG-TCNet demonstrated a notable classification accuracy of 77.35%.

Beyond EEGNet, researchers have introduced several other impactful methods for CNN-based classification of MI-EEG. Jia et al. introduce an innovative CNN-based framework that integrates spatial and temporal information processing to enhance the classification of motor imagery EEG signals^37^. The proposed model, featuring a time-contained spatial filtering (TSF) and a spatial–emporal analysis network (STAN), achieved average accuracies of 83.0% on the BCI IV-2a dataset and 88.0% on the BCI IV-2b dataset. The TSF-STAN model significantly surpasses existing approaches by effectively managing both spatial and temporal features. Amin et al. introduced MCNN, a fusion of multiple CNN models designed to harness diverse convolutional features for capturing both spatial and temporal elements from raw EEG data^29^. The MCNN achieved a classification accuracy of 75.72% on the BCI IV-2a dataset, and an impressive 95.4% on the High Gamma Dataset. Zhao et al. introduced a multi-branch 3D convolutional neural network (M3DCNN) tailored for MI classification, employing a novel approach to transform EEG signals into sequences of 2D arrays that retain the spatial distribution of sampling electrodes^30^. This innovative design allows the M3DCNN model to effectively capture both spatial and temporal dynamics of EEG data. For subject-specific analysis, the M3DCNN model demonstrated a commendable performance, achieving an accuracy of 71.02% and a kappa coefficient of 0.644 on the BCI IV-2a dataset. In the more challenging cross-subject evaluation, the model managed to attain an accuracy of 52.17% and a kappa coefficient of 0.453. Sakhavi et al. developed a classification framework for MI data that involves a novel temporal representation, created by modifying the FBCSP method, and a tailored CNN for classification^32^. This innovative approach significantly improved performance, outshining the existing method in the literature on the BCI competition IV-2a dataset with a 7% increase in average subject accuracy.

### Attention-based transformer architecture

The attention mechanism is an effort to emulate the human brain behavior of selectively focusing on a few significant elements while ignoring others. The self-attention mechanism has the intrinsic ability to evaluate global dependencies on very long sequences. A few studies attempted to adopt Transformer models for MI-EEG classification. Tao et al.^46^ implemented a modified version of the Transformer, known as the gated Transformer, for EEG signal analysis. This model employs a gating mechanism instead of traditional residual connections to learn feature representations along a sequence of embeddings. The gated Transformer demonstrated improved results in the PhysioNet dataset. Song et al. introduced an EEG decoding method focusing on self-attention mechanisms^47^. It starts with preprocessing and spatial filtering of EEG data, followed by applying attention transformations on the feature-channel dimension to emphasize key spatial features. The critical step involves segmenting the data temporally for attention processing, yielding distinct representations. Xie et al. crafted five Transformer-based models for MI-EEG classification, leveraging the Transformer model's capabilities and the spatial–temporal attributes of EEG signals^48^. These models achieved top classification accuracies of 83.31%, 74.44%, and 64.22% in two-, three-, and four-class motor-imagery tasks respectively during cross-individual validation on the PhysioNet dataset. They also highlighted that integrating positional embedding modules into the Transformer could further enhance EEG classification performance. However, these models bypass the importance of learning local features, which are crucial for EEG signal decoding.

Zhang et al. ingeniously integrated the Transformer with domain adaptation (DA) to address the variability in EEG signal distribution across different subjects. They introduced a cross-attention Transformer domain adaptive network, named MI-CAT. It achieves an average classification accuracy of 76.81% on the BCI IV-2a dataset and 85.26% on the BCI IV-2b dataset^24^. Drawing inspiration from ShallowConvNet and previously mentioned Transformer models, Song et al. introduced a compact convolutional Transformer named EEG Conformer, designed to capture both local and global features within a unified EEG classification framework^53^. The model utilizes ShallowConvNet for learning local features through one-dimensional temporal and spatial convolution layers. Additionally, a self-attention module is directly integrated to extract global correlations from the local temporal features. The EEG Conformer showcased notable accuracies of 78.66% on the BCI IV-2a dataset, 84.63% on the BCI IV-2b dataset, and 95.30% on the SEED dataset. Altaheri et al. developed an attention-based TCN (ATCNet) to enhance the accuracy of EEG-based motor imagery classification^54^. ATCNet utilizes a convolutional module, similar to EEGNet, for extracting local features from EEG trials, multi-head self-attention for emphasizing the most significant features in MI-EEG data, a temporal convolutional network for high-level temporal feature extraction, and a convolutional-based sliding window for efficient MI-EEG data augmentation. Through 10 runs executed on all subjects in the BCI IV-2a dataset, ATCNet achieved an average accuracy of 81.98% across all runs.

For cross-subject motor imagery BCIs, Keutayeva and Abibullaev explore the efficacy of attention mechanism-based models^55^. The study compares three models (ViT, Hybrid Spatial CNN + ViT, and Hybrid Temporal CNN + ViT) using the Leave-One-Subject-Out (LOSO) cross-validation method. Results highlight the effectiveness of the Hybrid Temporal CNN + ViT (HTCV) model on the BCI IV-2a dataset, though it underperforms on the BCI IV-2b dataset. This emphasizes the data size limitations affecting the performance variability of transformer models. Building upon reference^55^, Keutayeva and Abibullaev delve deeper into attention mechanisms, proposing the Spatio-Temporal CNN + ViT (st-CViT) model^56^. This study offers a comprehensive comparison of attention-based models, utilizing data augmentation techniques and employing nested LOSO for robust model selection. Their findings demonstrate that combining attention mechanisms with deep learning models significantly enhances the robustness and accuracy of subject-independent BCIs, particularly when leveraging enriched data environments.

Our research is an ongoing contribution to these works. Therefore, inspired by the works above, we propose the CTNet as an efficient backbone for MI-EEG decoding.

## Materials and methods

### Datasets

Two publicly available benchmark datasets, BCI IV-2a^57^ and IV-2b^58^ are used to evaluate the validity of our proposed end-to-end model. These two datasets are provided by the Graz University of Technology. The details of these two datasets, preprocessing and data augmentation are described below.

**Fig. 1:**
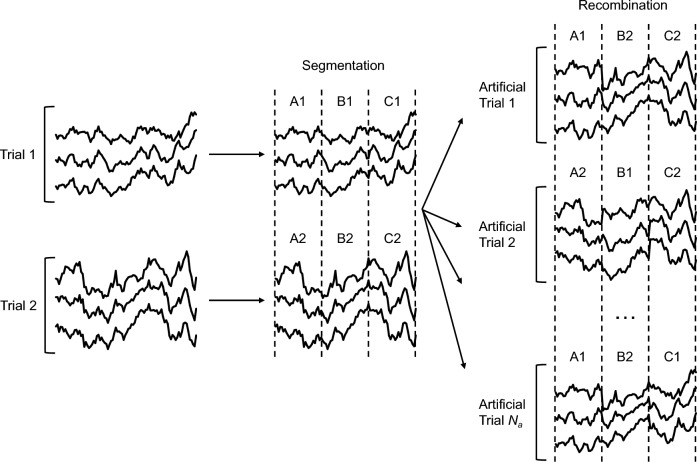
The principle of data augmentation.

1. BCI IV-2a dataset: The IV-2a dataset contains four MI tasks: left-hand, right-hand, both feet and tongue, which is available for download at the following link: [https://www.bbci.de/competition/download/competition_iv/BCICIV_2a_gdf.zip]. The dataset encompasses recordings from nine subjects (namely A01-A09), each participant contributed EEG data obtained through 22 Ag/AgCl electrodes with inter-electrode distances of 3.5 cm. The signals were sampled at 250 Hz, and the data underwent bandpass filtering from 0.5 to 100 Hz, with an additional 50 Hz notch filter activated to suppress line noise. Subjects participated in two sessions on separate days, with the first session allocated for training and the subsequent one for testing. Within each session, there were 288 trials, encompassing 72 trials per task. We used the temporal segment of^2,6^ seconds in our experiments. Each trial had the shape of a matrix of dimension (22, 1000).
2. BCI IV-2b dataset: The IV-2b dataset contains the recording of nine subjects (namely B01-B09), involving left-hand and right-hand MI activities, which is available for download at the following link: [https://www.bbci.de/competition/download/competition_iv/BCICIV_2b_gdf.zip]. Three bipolar recordings (C3, Cz, and C4) were recorded with a sampling frequency of 250 Hz. The recorded EEG signals were band-pass filtered from 0.5 to 100 Hz with a notch filter at 50 Hz set at the time of recording using signal acquisition hardware. Each subject participated in five sessions, where the first three sessions were for calibrating an EEG decoder and the rest sessions were for test purposes. There are about 400 trials and 320 trials in the training and test sets, respectively. We used the^3,7^ seconds of each trial in our experiments. Each trial was represented by a matrix with dimensions (3, 1000).
3. Preprocessing and Input Representation: Standardization, band-pass filtering and artifact removal are commonly employed methods to preprocess the raw EEG signals. In this study, we only applied the Z-score normalization to preprocess the EEG signals, ensuring a consistent data scale and enhancing the efficiency of model training. An MI-EEG raw trial $X_{i} \in {\text{R}}^{C \times T}$ consists of *C* electrode channels and *T* time samples. The standardization equation operation can be represented by the following equation:
  1 $$X_{i} ^{\prime} = \frac{{X_{i} - \mu }}{\delta }$$
  where $X_{i}{\prime} \in {\text{R}}^{C \times T}$ denotes the output of normalization, $\mu$ and $\delta$ represent the mean value and the standard deviation of raw trial $X_{i}$, respectively. The model's purpose is to associate each MI trial input, denoted as $X_{i}{\prime}$, with its corresponding category *y*_*i*_. This process involves utilizing a set of *m* MI trials labeled as $S = \left\{ {X_{i}{\prime} ,{ }y_{i} } \right\}_{i = 1}^{m}$, where ${ }y_{i} \in \left\{ {1,{ }...,{ }N} \right\}$, and *N* is the total number of defined categories for set *S*. For the BCI IV-2a dataset, *C* = 22 EEG channels, *T* = 1000 time samples, *N* = 4 MI categories, and *m* = 5184 MI trials. For the BCI IV-2b dataset, *C* = 3 EEG channels, *T* = 1000 time samples, *N* = 2 MI categories, and *m* = 6520 MI trials.
4. Data augmentation: The available EEG datasets for MI are limited, which can lead to overfitting when training deep learning models. To overcome this problem, an augmentation scheme needs to be introduced that may help in using a small amount of available data in an optimal way for training deep learning model. Existing data augmentation strategies include adding Gaussian white noise, cropping data, segmentation and recombination (S&R), and so on. In this study, we employ S&R in the time domain to augment the training set. Following ^59^, the augmentation process involves equally dividing each EEG training trial into several non-overlapping segments, followed by the generation of new artificial trials. The generation is accomplished by concatenating these segments in their original temporal order, each sourced from different but randomly chosen training trials belonging to the same category, thereby preserving the inherent time order. More formally, let us denote $\Omega \in \left\{ {X_{i}{\prime} } \right\}$, $i \in \left[ {1,{ }M} \right]$ as the set of *M* EEG trials that are available for training for a given category. Each training trial $X_{i}{\prime}$ is split into *K* continuous segments $X_{i}^{^{\prime}k} \epsilon R^{C \times T/K}$, $k\epsilon \left[ {1,K} \right]$. The generation of the artificial trial is represented by $\tilde{X}_{i}$ as $\tilde{X}_{i} = \left[ {X_{{R_{1} }}^{^{\prime}1} , X_{{R_{2} }}^{^{\prime}2} , ..., X_{{R_{K} }}^{^{\prime}K} } \right]$, where [*A*, *B, C*] represent the sequential concatenation of samples from segments A, B, and C, respectively. *R*_*k*_ is a randomly selected integer from the range [1, *M*]. This entire methodology is depicted and explained in detail in Fig. 1.

### Overall framework of proposed model

**Fig. 2:**
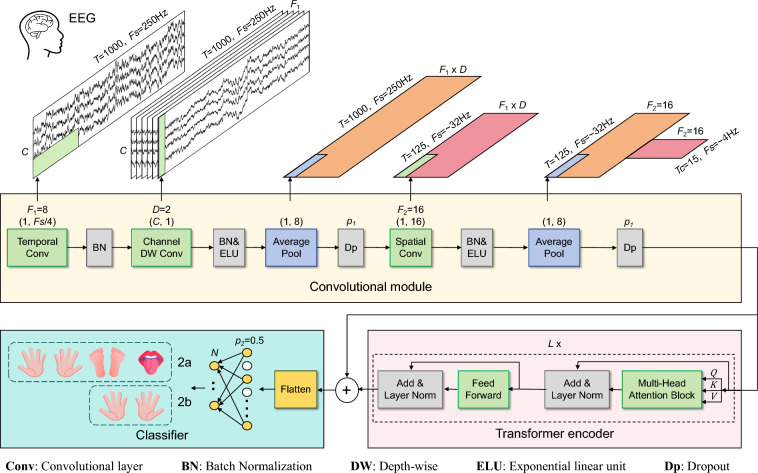
Proposed convolutional transformer network architecture for MI-EEG classification.

In this study, we have developed a convolutional transformer model for MI-EEG decoding, incorporating the MHA mechanism^45^. The overall framework of our model is depicted in Fig. 2. The framework facilitates an end-to-end classification of EEG trials, eliminating the need for exploring handcrafted feature extraction techniques. The proposed model consists of three main components: a convolutional module, a Transformer encoder module and a fully connected classifier. The convolutional module encodes low-level spatial–temporal information within the MI-EEG trial through three convolutional layers: a temporal convolution, a channel depth-wise convolution and a spatial convolution. The convolutional module processes the standardized EEG trial as input. It accounts for both the temporal continuity of the sampled EEG signals and the functional inter-connectivity among various electrode channels. The convolutional module outputs a higher-level temporal sequence representation. Following this, a Transformer encoder employs MHA to emphasize the most critical features within this sequence. Finally, the process concludes with a compact classifier, composed of two fully connected layers, which generates the decoding results.

### Convolutional module

The convolutional module in this study draws inspiration from EEGNet. We have designed the convolutional module by decomposing the two-dimensional convolution operator into two one-dimensional components: temporal and spatial convolution. Additionally, we also introduce depth-wise convolution. In contrast to EEGNet, we opt for one-dimensional convolution instead of separable convolution, resulting in improved performance.

The convolutional module consists of three convolutional layers, as shown in Fig. 2. The initial layer conducts a temporal convolution utilizing *F*_1_ filters with a size of (1, *K*_*C*1_), where *K*_*C*1_ represents the filter length along the time axis. This operation outputs *F*_1_ feature maps containing the EEG signal at different band-pass frequencies. The value of *K*_*C*1_ was configured to be one-fourth of the sampling rate (64). This choice enables the filters to capture temporal information associated with frequencies exceeding 4Hz. Subsequently, we employ a depth-wise convolution with *F*_2_ filters of size (*C*, 1) for spatial filtering, where *C* represents the number of EEG trial electrode channels (22 for the BCI IV-2a dataset and 3 for the BCI IV-2b dataset). This approach facilitates the learning of spatial filters corresponding to each temporal feature map, thereby efficiently extracting frequency-specific spatial filters. The depth parameter D, empirically set to 2, determines the number of spatial filters learned for each feature map. Hence, the output of the channel depth-wise convolution comprises *F*_1_ × *D* feature maps. Subsequent to the depth-wise convolution, an average pooling layer with a size of (1, *P*_1_) is employed to extract temporal information, down-sampling the signal's sampling rate by a factor of 8. This results in a reduced signal sampling rate of approximately 32 Hz. The third convolutional layer, a spatial convolution, comprises *F*_2_ filters of size (1, *K*_*C*2_). We set *K*_*C*2_ to 16 to decode motor imagery (MI) activities within a 500 ms window for data sampled at approximately 32 Hz. This is followed by a second average pooling layer of size (1, *P*_2_) for dimensionality reduction. The parameter *P*_2_ regulates the EEG sequential features sequence length (token size), which is then fed into the Transformer encoder.

All convolutional layers are succeeded by batch normalization (BN), which enhances the training process and alleviates overfitting. Following the second and third BN layers, exponential linear units (ELUs) are employed as the activation function to introduce non-linearity. The two average pooling layers are then followed by a dropout operation. For subject-specific classification, we set the dropout probability at 0.5 to help prevent overfitting when training with small sample sizes. For cross-subject classification, where the training sets are considerably larger, we reduced the dropout probability to 0.25. In the end, the convolution module generates feature maps $S \in {\text{R}}^{{T_{C} \times d}}$, where $T_{C}$ represents the length of the EEG trial's high-level feature representation, calculated as follows.

2 $$T_{C} = \frac{T}{{8 \times P_{2} }}$$

where *T* is the time samples of the raw EEG trial. Here, *d* denotes the number of feature channels, equivalent to *F*_2_, which is set to 16. Subsequently, all feature channels at each time point are fed as a token into the Transformer encoder.

### Transformer encoder

The Transformer network was initially introduced for machine translation, employing an encoder–decoder architecture with stacked MHA and position-wise fully connected feed forward block. Layer normalization (LN) and residual connections are incorporated to enhance the training efficiency and robustness of the model. In our study, focused on classification, we exclusively utilize the encoder block within the Transformer, comprising *L* layers in depth. Each layer contains two sub-layers: MHA mechanism and feed forward network. The overall architecture of the Transformer encoder is depicted in Fig. 2.

**Fig. 3:**
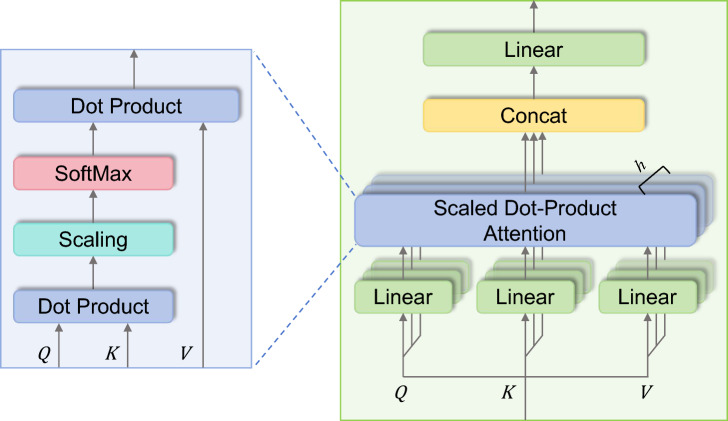
Multi-head attention.

The MHA mechanism is employed to capture global temporal dependencies in the high-level representation of EEG, thereby complementing the limited receptive field in the convolution module. The MHA comprises multiple self-attention layers known as heads, as illustrated in Fig. 3. The model transforms the input by multiplying *S* with three distinct weight matrices, thereby deriving three principal components: the queries $Q$, keys *K*, and values *V*.

$$Q_{i} = S W_{i}^{Q} \in {\text{R}}^{{T_{C} \times d_{k} }} , W_{i}^{Q} \in {\text{R}}^{{d \times d_{k} }}$$

3 $$K_{i} = SW_{i}^{K} \in {\text{R}}^{{T_{C} \times d_{k} }} , W_{i}^{K} \in {\text{R}}^{{d \times d_{k} }}$$

$$V_{i} = SW_{i}^{V} \in {\text{R}}^{{T_{C} \times d_{k} }} , W_{i}^{V} \in {\text{R}}^{{d \times d_{k} }}$$

where $W_{i}^{Q}$, $W_{i}^{K}$ and $W_{i}^{V}$ are the projection matrix of the query, key and value of the *i-*th head, respectively. The "Scaled Dot-Product Attention" mechanism calculates the dot products of the query with all keys, normalizes these products by dividing them by $\sqrt {d_{k} }$, and then applies a softmax function to determine the weights assigned to the values. The attention score *Z*_*i*_ of each attention head is calculated as follows:

4 $$Z_{i} = {\text{SA}}\left( {Q_{i} , K_{i} ,V_{i} } \right) = softmax\left( {\frac{{Q_{i} K_{i}^{T} }}{{\sqrt {d_{k} } }}} \right)V_{i} \in {\text{R}}^{{T_{C} \times d_{k} }}$$

The MHA enables the model to concurrently process and integrate information from various representation sub-spaces at different positions. By executing several SA operations in parallel, with each operation targeting distinct facets of the input, the MHA provides a comprehensive analysis of the input data. The outputs from these individual SA units are then coalesced through a linear transformation. This integration enables the model to effectively discern and encapsulate a broad spectrum of data dependencies, enhancing its representational capability.

$${\text{MHA}}\left( {Q,{ }K,{ }V} \right) = Concat\left( {Z_{1} ,{ }Z_{2} ,{ }...,{ }Z_{h} } \right)W^{O} \in {\text{R}}^{{T_{C} \times d_{k} }} , W^{O} \in {\text{R}}^{{hd_{k} \times d}}$$

where *h* denotes the number of heads. As a result of this mechanism within the MHA block, every node in the network acquires a global receptive field, enabling the model to capture and integrate information from across the entire input sequence. After processing through the MHA block, the output features are combined with the original input features *S* through a residual connection. Subsequently, the model employs LN to standardize each feature. The output of the MHA mechanism can be expressed as:

5 $$O = {\text{LN}}\left( {{\text{MHA}}\left( {Q,{ }K,{ }V} \right) + S} \right)$$

The following position-wise fully connected feed forward network is also conducted in a residual network form. It is applied to each position separately and identically. This sub-layer consists of two linear transformations with a Gaussian Error Linear Unit (GELU) activation and a dropout operation in between. The GELU activation function's formula is given by:

6 $${\text{GELU}}\left( x \right) = x{\Phi }\left( x \right)$$

where ${\Phi }$(*x*) is the cumulative distribution function of the standard Gaussian (normal) distribution, which can be expressed as:

7 $${\Phi }\left( x \right) = \frac{1}{2}\left[ {1 + {\text{erf}}\left( {\frac{x}{\sqrt 2 }} \right)} \right]$$

where erf(*x*) denotes the error function, which is a special function integral of the Gaussian distribution. Subsequently, LN is performed. Finally, the sum of the input feature and output feature is used as the output in a residual operation:

8 $$E = {\text{LN}}\left( {PF\left( O \right) + O} \right)$$

where $PF$ denotes the position-wise feed forward operation.

### Classifier module

In the classification block, the convolutional module and Transformer encoder's output features are added, enabling direct transmission of features extracted by the CNN to the classifier, and then flattened. Following this, a dropout operation is applied to mitigate overfitting and enhance generalization, with a dropout probability of 0.5. Finally, these processed features are fed into a fully connected layer comprising *N* units, where *N* represents the number of categories in the MI EEG classification task. Cross-entropy is used as the loss function for the entire model, as follows:

9 $${\mathcal{L}} = - \frac{1}{M}\mathop \sum \limits_{i}^{M} \mathop \sum \limits_{j}^{N} y_{ij} {\text{log}}\left( {\hat{y}_{ij} } \right)$$

where *M* is the number of the EEG trials, $y_{ij}$ is the true label for the *j*-th class in the *i*-th sample, and $\hat{y}_{ij}$ is the predicted probability for the *j*-th class in the *i*-th sample.

### Performance metric

To ensure a thorough evaluation, four widely recognized metrics are employed: accuracy, Cohen's Kappa, Hedges’ g and Wilcoxon p-value. Accuracy is calculated as:

10 $$Accuracy\left( {Acc} \right) = { }\frac{TP + TN}{{TP + TN + FP + FN}}$$

where *TP* and *TN* represent the correct positive sample number and the correct negative sample number predicted by the model, respectively, and *FP* and *FN* denote the false positive sample number and the false negative sample number predicted by the model, respectively. Cohen’s Kappa is calculated as follows:

11 $$\kappa { } = { }\frac{{P_{o} - P_{e} }}{{1 - P_{e} }}$$

where $P_{o}$ denotes the accuracy of the model, and $P_{e}$ represents the probability or accuracy of a random guess.

To assess the effect size of the Transformer module and data augmentation operation on model performance, we use Hedges' g. This metric is particularly suitable for small sample sizes and provides a more accurate estimate of effect size by correcting Cohen's d for sample size. Hedges' g is calculated as follows ^60^:

12 $$g = d \times \left( {1 - \frac{3}{{4\left( {n_{1} + n_{2} } \right) - 9}}} \right)$$

where *d* (Cohen's *d*) is calculated as:

$$d = \frac{{\overline{X}_{1} - \overline{X}_{2} }}{{\sqrt {\frac{{\left( {n_{1} - 1} \right)s_{1}^{2} + \left( {n_{2} - 1} \right)s_{2}^{2} }}{{n_{1} + n_{2} - 2}}} }}$$

where $\overline{X}_{1}$ and $\overline{X}_{2}$ are the means of the two groups being compared (e.g., with and without the Transformer module), *s*_1_ and *s*_2_ are the standard deviations of the two groups, and *n*_1_ and *n*_2_ are the sample sizes of the two groups, respectively. In this study, *n*_1_ and *n*_2_ are both equal to 9. Specifically, *g* around 0.2 suggests a small but potentially meaningful impact. *g* around 0.5 denotes a moderate impact that is likely to be of practical significance. *g* around 0.8 or higher indicates a large impact, which is very likely to be of substantial practical significance. These benchmarks help in interpreting the magnitude and importance of the effects in the context of experimental findings.

Additionally, we utilize the *p*-value derived from the paired Wilcoxon Signed-Rank Test to assess the statistical significance of differences between the proposed model and other state-of-the-art approaches. In this context, a *p*-value > 0.05 indicates the absence of a statistically significant difference. Conversely, a *p*-value < 0.05 (denoted as ‘*’) signifies a notable statistical difference, and a *p*-value < 0.01 (denoted as ‘**’) indicates a highly significant statistical difference.

### Training procedure

The training of our models was conducted on an Nvidia RTX3090 with 24 GB memory GPU. We utilized PyTorch, an open-source deep learning framework, on a workstation equipped with the Debian 11 operating system and an Intel Core i9-9820X CPU. Our analysis focused exclusively on EEG-channel data, and we chose to directly discard the three electrooculography (EOG) channels without engaging in any artifact removal procedures. The proposed model is evaluated using subject-specific and cross-subject. We have reserved 30% of the initial training set to function as a validation set. The model that demonstrated the minimum loss on the validation set was selected.

For subject-specific evaluations, we adhered to the data division scheme outlined in the competition guidelines. The following training configurations were adhered to: The Adam optimizer was employed, configured with a learning rate of 0.001, and *β*_1_ and *β*_2_ parameters were set to 0.5 and 0.999, respectively. The loss function used was categorical cross-entropy. We set the batch size at 288 and the number of training epochs at 1000. The dropout rate *p*_*1*_ is set to 0.5. Unless specified otherwise, the hyperparameters utilized across all experiments for two datasets are detailed in Table 1. These hyper-parameters were carefully selected following a series of preliminary experiments, aimed at achieving the best possible generalization of our model. We maintained consistency in these parameters for all subjects involved in the study to ensure uniformity and comparability of results.

**Table 1:** Global hype-parameters used for all subjects.

Convolutional module		Transfomer encoder module	
Temporal filters (*F*_1_)	8	Attention heads (*h*)	2
Kernel size (*K*_*c*1_)	64	Depth (*L*)	6
Depth multiplier (*D*)	2		
1st pooling size (*P*_1_)	8		
Dropout rate (*p*_*1*_)	0.5 or 0.25	Classifer module	
Spatial filters (*F*_2_)	16	Dropout rate (*p*_*2*_)	0.5
Kernel size (*K*_*c*2_)	16		
2nd pooling size (*P*_2_)	8		

For cross-subject evaluation, we employ the LOSO method. In this approach, we sequentially select one subject from the total of nine as the test subject, while aggregating the EEG data from the remaining subjects to form the training dataset. This process is repeated for each subject, thereby ensuring that the model is trained and evaluated on diverse subsets of data. The learning rate, batch size, and number of training epochs have been configured to 0.001, 512, and 600, respectively. The dropout rate *p*_*1*_ is set to 0.25.

## Experimental results

### Comparison with state-of-the-art approaches

In our study, we performed comprehensive subject-specific and cross-subject experiments and compared our method against several state-of-the-art approaches on the BCI IV-2a and IV-2b datasets. To ensure a relatively fair comparison, we reimplemented 4 prominent models (ShallowConvNet, DeepConvNet, EEGNet, and Conformer) based on their open-source code, maintaining uniform experimental conditions including data preprocessing, training and validation set splits, and data augmentation strategies. For these models, hyperparameters were configured to align with the specifics provided in their respective papers, except for the learning rate, batch size, and training epochs. These parameters were standardized across all models, including CTNet, to facilitate a fairer comparison.

#### Subject-specific classification

These excellent methods include ShallowConvNet, DeepConvNet, EEGNet and TSF-STAN, which are recognized for their remarkable CNN-based end-to-end performance in EEG datasets. We also assessed the performance of the Conformer, which effectively integrates local features captured by CNNs with global features processed by the Transformer encoder. Additionally, we conducted comparisons against MI-CAT, a domain adaptation network that leverages the Transformer’s self-attention and cross-attention mechanisms. The experimental results of TSF-STAN and MI-CAT adopt the data reported in their papers.

As shown in Table 2, our proposed model, CTNet, demonstrates robust performance. In the subject-specific experiments on the BCI IV-2a dataset, CTNet achieved an impressive average accuracy of 82.52%, which is only 0.48% less than the top-performing TSF-STAN model. CTNet recorded the highest classification accuracies for subjects A01, A03, and A04. Additionally, it exhibited the most consistent performance across all subjects, as evidenced by the lowest standard deviation of 9.61% and the highest Kappa score of 0.7670, indicating remarkable accuracy consistency among different subjects. The CTNet significantly surpassed the ShallowConvNet by 6.83% in accuracy (*p* < 0.01). Similarly, CTNet displayed comparable improvements over EEGNet. Moreover, CTNet's average classification accuracy exceeded that of DeepConvNet by 4.74%, Conformer by 4.86%, and MI-CAT by 5.71% (*p* < 0.05). These results collectively demonstrate the effectiveness of the CTNet, which encapsulates local features through CNN and global features via Transformer.

**Table 2:** Subject-specific classification accuracy (in percentage %) and Kappa of state-of-the-art algorithms on the BCI IV-2a dataset.

	A01	A02	A03	A04	A05	A06	A07	A08	A09	Average ± Std	*p*-value	Kappa
ShallowConvNet^+^^27^	82.64	55.21	92.01	74.31	72.92	59.72	81.60	83.33	79.51	75.69 ± 11.76	0.0020**	0.6759
DeepConvNet^+^^27^	82.29	44.79	90.63	76.04	**77.43**	**68.06**	**92.01**	83.33	85.42	77.78 ± 14.42	0.1934	0.7037
EEGNet^+^^28^	88.19	56.94	93.06	71.18	70.49	62.85	87.15	82.64	84.03	77.39 ± 12.47	0.0059**	0.6986
TSF-STAN^37^	88.3	**81.7**	92.2	77.6	63.3	67.5	90.0	**95.0**	**91.7**	**83.0** ± 11.4	0.7695	0.765
Conformer^+^^53^	85.07	48.96	91.32	78.47	75.00	65.28	87.85	87.15	79.86	77.66 ± 13.35	0.0742	0.7022
MI-CAT^24^	90.62	54.51	91.32	72.57	63.19	62.85	87.15	85.07	84.03	76.81 ± 13.80	0.0273*	0.692
CTNet (Proposed)	**90.97**	73.61	**96.53**	**84.72**	77.08	64.24	86.11	84.38	85.07	82.52 ± **9.61**	-	**0.7670**

^+^Reimplemented.

The bold font highlights the best result among the different methods.

Table 3 provides a comparison of subject-specific classification performance against several leading algorithms using the BCI IV-2b dataset. CTNet stands out in comparison, achieving an average accuracy of 88.49% and a Kappa score of 0.7697. It also shows the most consistent performance across different subjects, as indicated by the lowest standard deviation of 9.03%. Compared to ConvNet architectures, CTNet displayed significant improvements, outperforming ShallowConvNet by 3.36% (*p* < 0.01) and DeepConvNet by 3.28% (*p* < 0.01). CTNet's average classification accuracy surpasses that of EEGNet and TSF-STAN by 0.78% and 0.49%, respectively. Compared to the hybrid CNN and Transformer architectures of Conformer and MI-CAT, CTNet achieves higher accuracies by 2.62% (*p* < 0.05) and 3.21%, respectively.

**Table 3:** Subject-specific classification accuracy (in percentage %) and Kappa of state-of-the-art algorithms on the BCI IV-2b dataset.

	B01	B02	B03	B04	B05	B06	B07	B08	B09	Average ± Std	*p*-value	Kappa
ShallowConvNet^+^^27^	77.81	61.79	83.13	97.50	93.13	83.44	92.50	91.88	85.00	85.13 ± 10.74	0.0078**	0.7026
DeepConvNet^+^^27^	75.00	67.50	81.56	97.81	91.56	82.50	90.31	93.13	87.50	85.21 ± 9.56	0.0078**	0.7042
EEGNet^+^^28^	78.75	67.50	**85.94**	97.50	94.69	90.00	93.13	92.50	89.38	87.71 ± 9.33	0.293	0.7542
TSF-STAN^37^	86.1	**77.9**	67.6	98.5	91.7	95.8	91.7	90.8	**91.7**	88.0 ± 9.6	0.9102	–
Conformer^+^^53^	73.13	67.50	79.06	97.19	96.88	83.13	93.13	92.81	90.00	85.87 ± 10.73	0.0117*	0.7174
MI-CAT^24^	**86.11**	65.97	61.46	**98.26**	93.75	89.24	86.11	**95.69**	90.97	85.28 ± 12.93	0.5703	0.706
CTNet (Proposed)	78.75	71.07	84.38	97.19	**97.81**	87.81	**94.06**	94.69	90.63	**88.49 ± 9.03**	–	**0.7697**

^+^Reimplemented.

The bold font highlights the best result among the different methods.

**Fig. 4:**
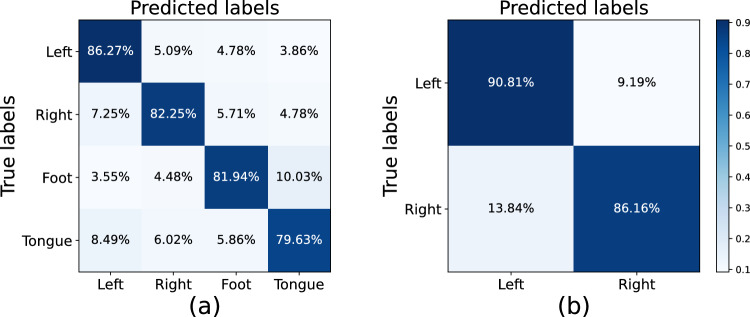
Average confusion matrices of the proposed CTNet, (**a**) the BCI IV-2a dataset (**b**) the BCI IV-2b dataset.

The average confusion matrices for CTNet are depicted in Fig. 4. Figure 4a presents the confusion matrix for the BCI IV-2a dataset, indicating that the left hand imagery is the easiest to recognize with an accuracy of 86.27%, while tongue imagery proves the most challenging with an accuracy of 79.63%. The most frequent misclassification occurred when imagined foot movements were incorrectly classified as imagined tongue movements, with a misclassification rate of 10.03%. Furthermore, Fig. 4b displays the average confusion matrix for the BCI IV-2b dataset, highlighting that the accuracies for decoding left and right hand imagery are 90.81% and 86.16%, respectively. The proportion of misclassifications where imagining left-hand movements were incorrectly identified as right-hand movements reached 9.19%, while instances of imagining right-hand movements being misclassified as left-hand movements stood at 13.84%.

#### Cross-subject classification

Achieving widespread utilization of BCI systems necessitates surmounting the challenge of individual differences. A pivotal criterion for the success of MI EEG classification algorithms is their ability to generalize across subjects. To assess this capability, we undertook cross-subject evaluations using the LOSO methodology, with findings detailed in Table 4 on the BCI IV-2a dataset and Table 5 on the BCI IV-2b dataset.

**Table 4:** Cross-subject classification accuracy (in percentage %) and Kappa of state-of-the-art algorithms on the BCI IV-2a dataset.

	A01	A02	A03	A04	A05	A06	A07	A08	A09	Average ± Std	Kappa
ShallowConvNet^+^^27^	66.84	46.53	67.53	52.26	34.38	39.76	65.45	71.18	66.84	56.75 ± 13.77	0.4234
DeepConvNet^+^^27^	68.58	**47.40**	78.99	52.26	**50.87**	**41.84**	**69.44**	71.70	60.24	**60.15 ± 12.71**	**0.4686**
EEGNet^+^^28^	**69.79**	42.01	**79.51**	50.87	35.76	37.15	65.80	67.36	63.37	56.85 ± 15.82	0.4246
Conformer^+^^53^	68.75	37.33	69.62	43.58	29.51	35.24	58.33	**74.48**	63.89	53.41 ± 17.08	0.3789
CTNet (Proposed)	69.27	43.92	79.34	**55.38**	43.92	36.11	65.10	70.66	**64.06**	58.64 ± 14.61	0.4486

^+^Reimplemented.

The bold font highlights the best result among the different methods.

**Table 5:** Cross-subject classification accuracy (in percentage %) and Kappa of state-of-the-art algorithms on the BCI IV-2b dataset.

	B01	B02	B03	B04	B05	B06	B07	B08	B09	Average ± Std	Kappa
ShallowConvNet^+^^27^	74.03	63.53	59.72	82.84	82.43	**80.97**	74.86	72.37	77.78	74.28 ± 8.13	0.4856
DeepConvNet^+^^27^	74.03	65.15	63.47	80.81	82.70	74.86	**81.39**	**76.32**	**77.92**	75.18 ± 6.84	0.5037
EEGNet^+^^28^	74.44	69.26	62.36	80.41	**83.24**	75.56	79.86	73.55	77.50	75.13 ± 6.35	0.5026
Conformer^+^^53^	71.39	62.35	65.28	**82.97**	80.41	69.31	75.00	**76.32**	78.61	73.52 ± 6.96	0.4703
CTNet (Proposed)	**76.25**	**71.03**	**66.39**	81.76	83.11	77.22	79.17	73.56	**77.92**	**76.27 ± 5.26**	**0.5252**

^+^Reimplemented.

The bold font highlights the best result among the different methods.

As indicated by Table 4, on the BCI IV-2a dataset, CTNet achieved an average classification accuracy of 58.64%, which is second only to DeepConvNet by a margin of 1.51%. CTNet also ranks second in terms of standard deviation and Kappa value, at 14.61% and 0.4486, respectively. Furthermore, CTNet's average accuracy surpasses that of ShallowConvNet, EEGNet, and Conformer by 1.89%, 1.79%, and 5.23% (p < 0.05) respectively. As shown in Table 5, on the BCI IV-2b dataset, CTNet achieved the highest average classification accuracy of 76.27%. It also recorded the smallest standard deviation at 5.26% and the highest Kappa value of 0.5252. CTNet performed exceptionally well for subjects B01, B02, B03, and B09. Compared to purely CNN-based models like ShallowConvNet, DeepConvNet, and EEGNet, CTNet's average accuracy was higher by 1.99%, 1.09%, and 1.14%, respectively, and 2.75% higher than the CNN and Transformer hybrid architecture, Conformer. The funding from Tables 4 and 5 collectively underscores CTNet's superior generalization capacity.

### Ablation study

**Fig. 5:**
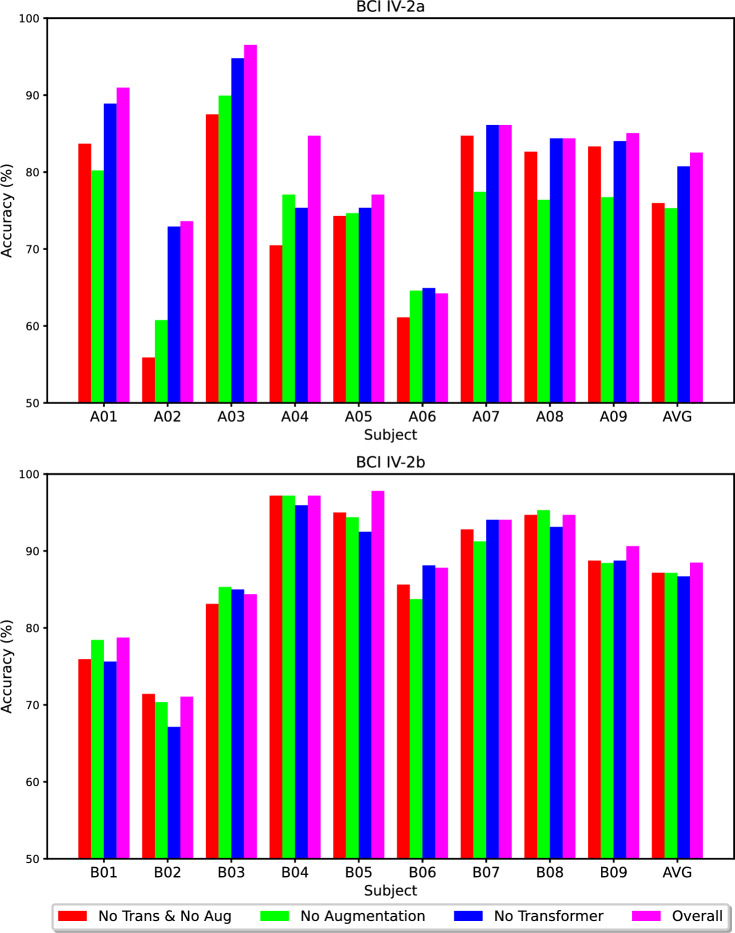
Ablation study on data augmentation and Transformer encoder module on BCI IV-2a (Top) and BCI IV-2b (Bottom).

A key advancement of CTNet over CNN-based methods is the incorporation of a Transformer encoder module, which employs MHA to learn global representations of high-level features in EEG trials. Additionally, data augmentation may also contribute to the final decoding results. Consequently, we performed ablation studies on subject-specific classification experiments within the BCI IV-2a and IV-2b datasets. Our ablation experiments involved individually removing S&R data augmentation, individually removing the Transformer, and removing both data augmentation and the Transformer concurrently. The results of these experiments are illustrated in Fig. 5. The effect sizes for the ablation experiments are presented in Table 6.

**Table 6:** Effect sizes for various ablation experiments.

Condition	BCI IV-2a (Hedges' g)	BCI IV-2b (Hedges' g)
WA + WT vs. WA + NT	0.179	0.184
NA + WT vs. NA + NT	− 0.063	− 0.002
WT + WA vs. WT + NA	0.758	0.143
NT + WA vs. NT + NA	0.441	− 0.049
WA + WT vs. NA + NT	0.595	0.139

WA represents with augmentation, WT represents with the Transformer module, NA represents no augmentation, and NT represents no Transformer module.

For the BCI IV-2a dataset, ablation experiments are depicted in the upper subplot of Fig. 5. It is evident from Fig. 5 that for the majority of subjects, removing either the Transformer module, data augmentation, or both, significantly diminishes the recognition accuracy. Specifically, excluding the Transformer alone resulted in a noticeable decrease in recognition accuracy for most subjects (A01-A05 and A09), with a particularly pronounced drop of 9.37% for subject A04; however, participants A07 and A08 did not experience any change in accuracy, and subject A06 actually showed a slight increase of 0.69%. Overall, the removal of the Transformer module led to an average notable reduction in model accuracy by 1.77% (*p* < 0.05, g = 0.179). The effect size for the Transformer module, as measured by Hedges' g, is 0.179. This indicates a small effect size, suggesting that the inclusion of the Transformer module has a positive impact on model performance.

When data augmentation was removed alone, all subjects except A06 exhibited a marked decrease in accuracy, averaging a significant decline of 7.21% (*p* < 0.01, g = 0.758). Given that Transformer models typically require substantial amounts of training data, this result indicates a medium to large effect size. This underscores the critical role of data augmentation in maximizing the performance benefits of the Transformer module, which typically requires substantial training data. Simultaneously removing both the Transformer and data augmentation resulted in an average accuracy reduction of 6.55% across all participants (*p* < 0.01, g = 0.595), highlighting the significant contributions of both components to recognition accuracy.

Additionally, we observed that in the absence of data augmentation, due to the limited training data, incorporating the Transformer module could actually decrease the recognition accuracy for four subjects (especially a 7.29% drop for A07), with an average decline of 0.66%.

For the BCI IV-2b dataset, as shown in the lower subplot of Fig. 5, the impact of removing the Transformer module mirrors that observed in the BCI IV-2a dataset, with a notable decrease in recognition accuracy across most participants, averaging a reduction of 1.79% (*p* < 0.05, *g* = 0.184). Removal of either data augmentation or both modules did not reduce accuracy as drastically as in the BCI IV-2a dataset, with average decreases of 1.33% (*g* = 0.143) and 1.32% (*p* < 0.05, *g* = 0.139) respectively. Furthermore, without data augmentation, the inclusion of the Transformer does not enhance the model's average recognition accuracy.

### Effect of hyper-parameters

In this section, we meticulously assess the influence of various critical hyper-parameters on the performance of the model in subject-specific classification. These parameters encompass the length of the high-level EEG features (token size) inputted into the Transformer module, the number of self-attention heads, and the depth of the Transformer encoder architecture.

**Fig. 6:**
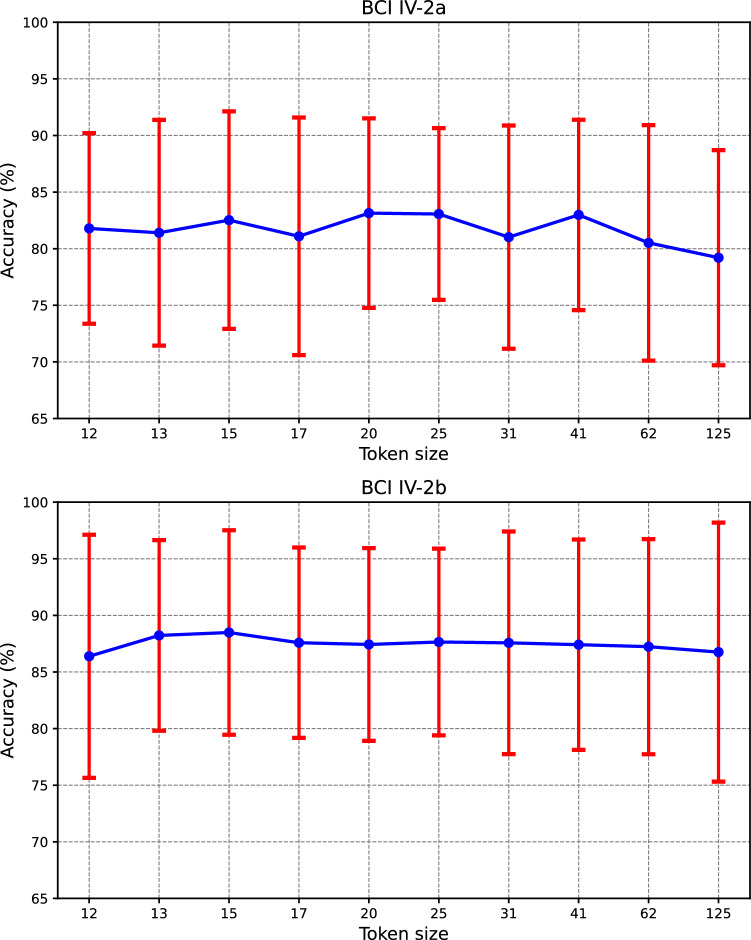
Performance of CTNet with different token sizes on BCI IV-2a (Top) and BCI IV-2b (Bottom).

Token size *T*_*c*_, as determined by the second average pooling kernel, plays a critical role in the effectiveness of the Transformer encoder. Excessively large pooling kernel sizes may overly smooth temporal features, obliterating valuable details, whereas too small sizes might render the model susceptible to local noise disturbances. This necessitates a delicate balance in selecting the convolution module's second pooling size. To address this, we evaluated the effects of varying pooling sizes on model performance within the BCI IV-2a and IV-2b datasets to identify an optimal size that enables the model to discern global features without succumbing to local noise interference, as illustrated in Fig. 6. The upper and lower subplots in Fig. 6 respectively illustrate the impact of different token sizes on recognition accuracy within the BCI IV-2a and BCI IV-2b datasets. Token size *T*_*c*_ ranged from 12 to 125. It is evident from Fig. 6 that both excessively large and small token sizes yield suboptimal performance. On the BCI IV-2a dataset, the optimal average accuracy is achieved at a token size of 20 (*P*_2_ = 6), reaching 83.14%. This is significantly better than the models with token sizes of 12 (*P*_2_ = 10) and 125 (*P*_2_ = 1), which are 3.94% (*p* < 0.01) and 1.35% (*p* < 0.01) less effective, respectively. Similarly, on the BCI IV-2b dataset, the best average accuracy is observed at a token size of 15 (*P*_2_ = 8), amounting to 88.49%. This surpasses the performance at token sizes of 12 and 125 by 2.01% (*p* < 0.05) and 1.73%, respectively. Hence, opting for a relatively larger pooling parameter *P*_2_, which results in comparatively smaller tokens, allows for more efficient utilization of EEG's local features, thereby enhancing the Transformer's capability to aggregate global features.

**Fig. 7:**
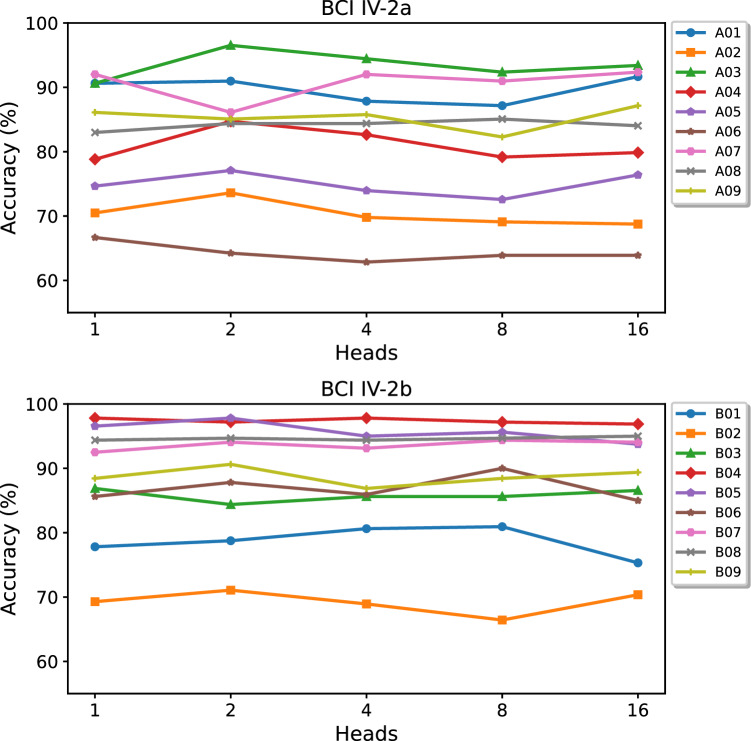
Performance of CTNet with different heads of self-attention on BCI IV-2a (Top) and BCI IV-2b (Bottom).

The number of heads is a critical parameter in the Transformer encoder, which leverages the MHA mechanism. MHA enables the model to process information concurrently, with each head concentrating on a distinct facet of the input sequence. Our research investigates the impact of varying the number of heads, as illustrated in Fig. 7, where we explore a range of head numbers *h* from 1 to 16. The upper and lower subplots in Fig. 7 depict the effects of varying numbers of attention heads on the model's performance across the BCI IV-2a and BCI IV-2b datasets, respectively. It is evident from the figure that the accuracy for the same subject fluctuates across different numbers of attention heads in the CTNet, indicating the model's sensitivity to the number of heads. CTNet demonstrates greater sensitivity to the number of heads in the BCI IV-2a dataset compared to BCI IV-2b. Overall, across both the BCI IV-2a and IV-2b datasets, the CTNet models equipped with two attention heads yield the best performance. On the BCI IV-2a dataset, the model with two heads surpasses those with 1, 4, 8, and 16 heads in terms of average recognition accuracy by 1.08%, 1.00%, 2.23%, and 0.58%, respectively. Similarly, on the BCI IV-2b dataset, the model with two heads outperforms those with 1, 4, 8, and 16 heads in terms of average recognition accuracy by 0.79%, 0.90%, 0.35%, and 1.12%, respectively. The Kappa index exhibits a similar pattern of results.

**Fig. 8:**
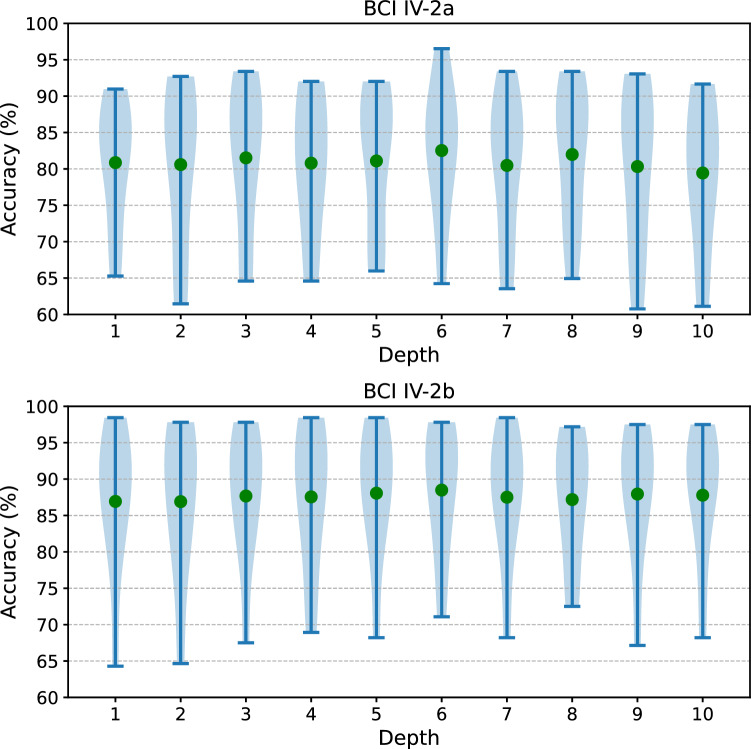
Performance of CTNet with different depth of Transformer encoder on BCI IV-2a (Top) and BCI IV-2b (Bottom), green point denotes mean.

Depth *L* is a critical factor that influences the fitting capabilities of the Transformer model. We explored the effects of varying depth levels on the CTNet by incrementally increasing the number of Transformer encoder blocks from 1 to 10, as depicted in Fig. 8. The upper and lower subplots in this figure respectively illustrate the impact of Transformer depths on the CTNet model performance across the BCI IV-2a and BCI IV-2b datasets. It is evident that the distributions of recognition accuracy for different subjects' MI intentions vary with changes in the Transformer depth. Specifically, for the BCI IV-2a dataset, the model featuring a Transformer with a depth of 6 layers achieves the highest average recognition accuracy, reaching 82.52%, which is 3.09% higher than that of the model with a 10-layer Transformer. Similarly, in the BCI IV-2b dataset, a Transformer with 6 layers yields the highest average recognition accuracy at 88.49%, surpassing that of the 2-layer depth Transformer by 1.58% (p < 0.05).

### Visualization of feature distributed

**Fig. 9:**
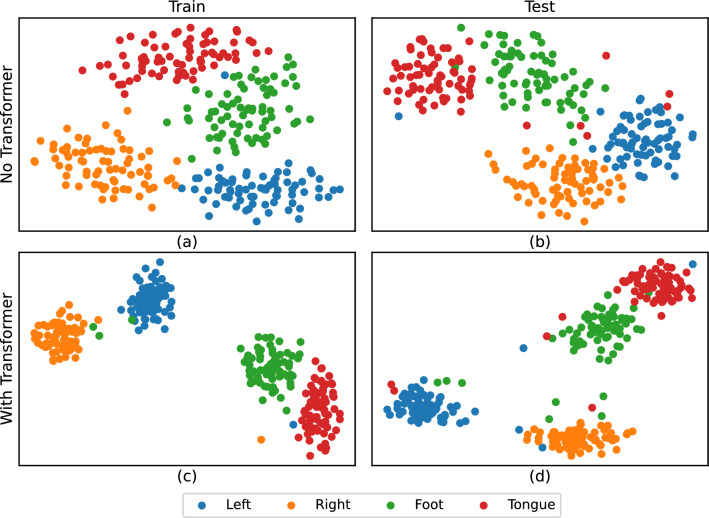
Two-dimensional t-SNE visualization of the features.

To demonstrate the capabilities of the proposed CTNet, we embarked on visualizing its feature extractions. Utilizing t-distributed stochastic neighbor embedding (t-SNE), a renowned technique for dimensionality reduction and visualization, we aimed to evaluate the discriminative capacity of the features extracted by our network. Figure 9 presents the visualization of feature distributions for subject A03 on subject-specific classification, comparing scenarios with and without the integration of the Transformer encoder module in both training and test datasets. Notably, the model's performance without employing the Transformer module, as depicted in Fig. 9a,b, showcases a relatively small inter-class distance among the four categorized features and a larger intra-class variance. Conversely, with the inclusion of the Transformer module, as illustrated in Fig. 9c,d, there is a marked increase in the inter-class separation and a substantial reduction in the intra-class distance. This outcome further corroborates that the fusion of CNN and the Transformer module substantially amplifies the discriminative capability of the features.

## Discussion

CNN-based methodologies have demonstrated efficacy in MI-EEG classification, chiefly due to CNN's robust capability in local feature extraction. Nonetheless, CNNs typically possess a limited receptive field, potentially impeding their ability to capture global feature dependencies. The Transformer model's self-attention mechanism effectively captures long-distance dependencies within data, facilitating a comprehensive understanding of the entire input sequence. This feature is particularly critical in MI-EEG signal processing, where MI tasks involve complex cortical coordination that often spans extensive intervals in the time series. The Transformer's self-attention mechanism is essential for understanding and analyzing complex activity patterns across multiple time points. Additionally, the Transformer can dynamically adjust its focus, applying weighted attention to critical signal features within MI-EEG data, such as specific frequency band rhythm changes, thereby significantly enhancing the model's sensitivity to key information and accuracy in decoding. Based on these insights, we introduce the CTNet model, which combines the CNN’s capability for local feature extraction with the Transformer’s ability to process global information, offering substantial advantages in the decoding of MI-EEG signals. The efficacy of the CTNet has been validated through subject-specific and cross-subject classification experiments conducted on the BCI IV-2a and BCI IV-2b datasets.

### Discussion on subject-specific classification

In our study, we compare CTNet with leading algorithms based solely on CNN architectures (ShallowConvNet, DeepConvNet, EEGNet, TSF-STAN) and those combining CNN and Transformer frameworks (Conformer and MI-CAT) for subject-specific MI-EEG decoding. We reimplemented the models from the open-source code of ShallowConvNet, DeepConvNet, EEGNet, and Conformer to ensure a fair comparison under identical experimental conditions. To conduct a thorough comparison of these state-of-the-art algorithms, we delve deeper into four aspects: data preprocessing, data augmentation strategies, model architecture, and the quantity of trainable network parameters, with comparative results presented in Table 7. In 2017, inspired by the FBCSP algorithm, Schirrmeister et al. introduced the notable ShallowConvNet and DeepConvNet models. ShallowConvNet, employing just two one-dimensional convolutions (temporal and spatial), achieved notable results, with average accuracies and Kappa values of 75.69% and 0.6759 on the BCI IV-2a dataset, and 85.13% and 0.7026 on the BCI IV-2b dataset, respectively. DeepConvNet, building upon ShallowConvNet by adding three convolution-pooling blocks, improved performance on both datasets, particularly achieving a 2.09% and 0.08% increase in average accuracy and Kappa values on the BCI IV-2a dataset. The cost, however, was an increase of over 200,000 in the number of trainable parameters. In 2018, Lawhern et al. proposed the advanced EEGNet model. This model introduced depth-wise and separable convolutions, significantly reducing the quantity of trainable parameters to 2.9k for the BCI IV-2a dataset and 2.1k for the BCI IV-2b dataset, effectively mitigating overfitting. Among all compared models, EEGNet had the fewest parameters, which greatly aids in reducing training times and deploying the model on memory-constrained devices. EEGNet's average recognition accuracies on the BCI IV-2a and BCI IV-2b datasets were 1.70% and 2.58% higher than those of ShallowConvNet, with Kappa values also improving by 0.0227 and 0.0516, respectively. In 2022, Jia et al. introduced the TSF-STAN model. This model initially leverages time-contained spatial filtering for data preprocessing to increase the inter-category difference of EEG signals while preserving temporal features; it then utilizes a CNN-based spatial–temporal analysis network to further exploit discriminative spatial and temporal features and classify different EEG categories in an end-to-end process. Even without data augmentation, TSF-STAN's average recognition accuracies on the BCI IV-2a and BCI IV-2b datasets were 5.61% and 0.29% higher than those of EEGNet employing data augmentation. The performance might further improve if TSF-STAN utilized data augmentation. Ablation studies by Jia et al. also revealed that without the TSF preprocessing step, using only the STAN network would decrease the average recognition accuracy and Kappa value on the BCI IV-2a dataset by 17.3% (*p* < 0.01) and 0.2260, underscoring the significant performance boost provided by the STF preprocessing step. When compared to CTNet using data augmentation, TSF-STAN showed a 0.48% higher recognition accuracy on the BCI IV-2a dataset, while CTNet had a 0.49% higher accuracy on the BCI IV-2b dataset. Compared to the without data augmented CTNet and STAN model, our CTNet model's average recognition accuracy and Kappa value were higher by 9.61% and 0.1318, respectively.

**Table 7:** Comparative analysis of state-of-the-art algorithms for subject-specific classification.

Algorithms	Preprocessing	Augmentation	Architecture	BCI IV-2a			BCI IV-2b		
				Parameters	Accuracy	Kappa	Parameters	Accuracy	Kappa
ShallowConvNet^+^^27^	standardization	S & R	CNN	46.1 k	75.69	0.6759	10.8 k	85.13	0.7026
DeepConvNet^+^^27^	standardization	S & R	CNN	283.3 k	77.78	0.7037	268.6 k	85.21	0.7042
EEGNet^+^^28^	standardization	S & R	CNN	**2.9 k**	77.39	0.6986	**2.1 k**	87.71	0.7542
TSF-STAN^37^	TSF		CNN	105.0 k	**83.0**	0.765	104.5 k	88.0	–
Conformer^+^^53^	standardization	S & R	CNN + Transformer	166.1 k	77.66	0.7022	130.8 k	85.87	0.7174
MI-CAT^24^	BF + EMS		CNN + Transformer + DA	97.5 k	76.81	0.692	80.3 k	85.28	0.706
CTNet (Proposed)	standardization	S & R	CNN + Transformer	25.7 k	82.52	**0.7670**	24.9 k	**88.49**	**0.7697**
STAN^37^			CNN	105.0 k	65.7	0.539	104.5 k	–	–
CTNet(Proposed)	standardization		CNN + Transformer	25.7 k	75.31	0.6708	24.9 k	87.16	0.7432

^+^ Reimplemented. The bold font highlights the best result among the different methods.

Conformer and MI-CAT are exemplary models for decoding MI-EEG, utilizing a hybrid architecture that combines CNN and Transformer technologies. In 2023, combining the local feature extraction capabilities of ShallowConvNet with the global modeling strength of the Transformer, Song et al. proposed the Conformer model. On the BCI IV-2a and IV-2b datasets, the average recognition accuracies of the Conformer model were 1.97% and 0.74% higher than those of ShallowConvNet. This also demonstrates that incorporating a Transformer to globally model the high-level features extracted by CNNs can enhance the model's ability to recognize MI-EEG signals. Correspondingly, the model's trainable parameter number also increased by approximately 0.12 million. Our CTNet model, inspired by both Conformer and EEGNet, is designed to achieve high recognition accuracy while maintaining a smaller trainable parameter number, thus reducing overfitting and enhancing the model's generalization capability. In 2023, Zhang and colleagues proposed the MI-CAT model to address the inter-subject variability of EEG signals. MI-CAT employs a temporal-spatial CNN to learn feature representations from paired EEG data, followed by two domain-related attention blocks that preserve domain-dependent information. It then utilizes the Transformer’s self-attention and cross-attention mechanisms to facilitate feature interaction and resolve differential distributions across different domains. Additionally, MI-CAT uses bandpass filtering (BF) and exponential moving standardization (EMS) for data preprocessing. Without data augmentation, MI-CAT achieved remarkable average recognition accuracies of 76.81% and 85.28% on the BCI IV-2a and IV-2b datasets, respectively, with Kappa values of 0.692 and 0.706. In comparison with the CTNet model, which did not use data augmentation, MI-CAT exhibited a 1.50% higher average recognition accuracy on the BCI IV-2a dataset, while CTNet performed 1.88% better on the BCI IV-2b dataset. This shows that the recognition accuracy of CTNet and MI-CAT models is comparable. However, MI-CAT has over 55,000 more trainable parameters than CTNet.

In summary, compared to state-of-the-art methods such as ShallowConvNet, DeepConvNet, EEGNet, TSF-STAN, Conformer, and MI-CAT, the CTNet model is relatively small yet achieves comparable decoding accuracy to the TSF-STAN method on both the BCI IV-2a and IV-2b datasets. Specifically, CTNet's accuracy is higher than other state-of-the-art methods by 4.74% to 6.83% on the BCI IV-2a dataset and by 0.78% to 3.36% on the BCI IV-2b dataset. Notably, while TSF-STAN utilizes a complex TSF data preprocessing method, CTNet employs a simple standardization process, greatly simplifying the preprocessing pipeline. TSF-STAN's complex TSF preprocessing requires substantial computation, whereas CTNet's straightforward standardization process reduces computational complexity and resource demands. Achieving high accuracy without sacrificing performance highlights the practical advantages of our approach. In practical applications, reducing computational complexity can lead to shorter processing times, lower power consumption, and easier deployment, particularly in resource-constrained environments. Therefore, achieving accuracy comparable to the state-of-the-art TSF-STAN, combined with our simpler preprocessing pipeline, underscores the practical significance of our method.

### Discussion on cross-subject classification

In cross-subject MI-EEG decoding comparative analysis, we utilized the LOSO cross-validation method under consistent experimental conditions to compare CTNet with ShallowConvNet, DeepConvNet, EEGNet, and Conformer. On the BCI IV-2a dataset, the recognition accuracies of Conformer, ShallowConvNet, EEGNet, CTNet, and DeepConvNet progressively improved. DeepConvNet, the best-performing model, achieved a recognition accuracy 1.51% higher than the second-best, CTNet. On the BCI IV-2b dataset, accuracies improved sequentially for Conformer, ShallowConvNet, EEGNet, DeepConvNet, and CTNet, with the top-performing CTNet surpassing the second-best best, DeepConvNet, by 1.09%. These results clearly demonstrate CTNet's effectiveness in solving cross-subject MI-EEG decoding challenges. Additionally, Chowdhury et al. introduced EEGNet Fusion V2^31^, which integrates five distinct branches of EEGNet with varying hyperparameters, achieving average recognition accuracies of 74.3% and 84.1% in cross-subject MI-EEG decoding on the BCI IV-2a and IV-2b datasets, respectively. The fusion strategy of EEGNet Fusion V2 potentially offers richer feature representations and decision boundaries, mitigating the risk of overfitting or underfitting through an ensemble learning approach. Unlike CTNet, which employs a LOSO cross-validation strategy widely used in BCI research, EEGNet Fusion V2 utilizes a session-based division strategy: one session for training and another for testing on the BCI IV-2a dataset, and three sessions for training with the remaining two for testing on the BCI IV-2b dataset. This strategy likely reduces differences between training and testing data, as data from the same subject across different sessions tend to be more similar. This session-based approach might better adapt to individual subject characteristics, thereby enhancing accuracy during testing. Consequently, EEGNet Fusion V2 significantly outperforms CTNet in terms of accuracy, drawing attention to the merits of utilizing multiple branches with unique hyperparameters for broader feature extraction from EEG data, given the significant variability among different subjects.

For cross-subject MI-EEG decoding, Keutayeva and Abibullaev proposed the HTCV model^55^ and later the st-CViT model^56^. Both models employed S&R data augmentation strategies and LOSO cross-validation methods. HTCV is tested on the BCI IV-2a and BCI IV-2b datasets for decoding left and right hand movements, while st-CViT is evaluated on the BCI IV-2a, BCI IV-2b, Weibo, and Physionet datasets. Additionally, reference^56^ also tests the performance of st-CViT using the nested LOSO strategy, which enhances the reliability of their model evaluation.

From an architectural perspective, CTNet, HTCV, and st-CViT are hybrid models combining CNN and Transformer. In terms of decoding accuracy for the same classification task on the BCI IV-2b dataset, with the same data augmentation strategy and LOSO cross-validation evaluation method, the results are compared in Table 8. As shown in Table 8, CTNet outperforms HTCV and st-CViT in terms of average classification accuracy for binary classification on the BCI IV-2b dataset by 5.27% and 1.54%, respectively, with the smallest standard deviation of 5.26%. This superior performance can be attributed to CTNet's EEGNet-like CNN structure, which offers better local spatiotemporal feature extraction capabilities. Furthermore, CTNet effectively integrates features extracted by the CNN with those encoded by the Transformer encoder through residual connections, thus leveraging both the local feature extraction capability of CNNs and the global feature encoding capacity of Transformers. Despite CTNet's higher overall accuracy, it must be acknowledged that HTCV outperforms CTNet for individual subjects B04, B08, and B09, and st-CViT achieves higher decoding accuracy for subjects B01, B04, B06, B08, and B09. This discrepancy may be due to the individual variability in EEG signals among different subjects, which might require different feature extraction and classification strategies that are better handled by HTCV and st-CViT in these specific cases.

**Table 8:** Comparison of decoding accuracy between CTNet, HTCV, and st-CViT models on the BCI IV-2b dataset.

Models / Subjects	B01	B02	B03	B04	B05	B06	B07	B08	B09	Average ± Std
HTCV^55^	68	60	54	**85**	75	72	68	78	79	71 ± 9
st-CViT^56^	**77.36**	59.71	58.61	83.51	76.89	**80.42**	75.42	**78.82**	**81.81**	74.73 ± 8.65
CTNet (Proposed)	76.25	**71.03**	**66.39**	81.76	**83.11**	77.22	**79.17**	73.56	77.92	**76.27** ± **5.26**

The bold font highlights the best result among the different methods.

### Discussion on ablation study

Ablation studies demonstrate that in the task of decoding subject-specific MI-EEG, the S&R data augmentation method contributes to improved accuracy on both datasets, with a significant impact observed on the BCI IV-2a dataset. Furthermore, the removal of the Transformer module in the presence of data augmentation resulted in a notable decrease in recognition accuracy by 1.77% (*p* < 0.05) on the BCI IV-2a dataset and 1.79% (*p* < 0.05) on the BCI IV-2b dataset. This suggests that the Transformer module plays a notable role in harnessing the enriched data environment provided by augmentation techniques. The Transformer’s self-attention mechanism has the unique capability to process the entire input features sequence simultaneously, allowing it to capture global dependencies that are vital for understanding complex EEG patterns. This capability allows for a more nuanced understanding of EEG signals, particularly beneficial for tasks like MI where temporal dynamics are crucial. Furthermore, the global processing is particularly beneficial in datasets enriched through data augmentation, as it helps the model to generalize better across varied yet synthetically expanded data.

The effect size analysis provides further insights into the contributions of the Transformer module and data augmentation. For the BCI IV-2a and IV-2b datasets, when data augmentation was applied, adding the Transformer module resulted in effect sizes (Hedges' g) of 0.179 and 0.184, respectively. This suggests a small but positive impact of the Transformer module when data augmentation is utilized, highlighting its ability to enhance model performance by capturing global dependencies in the data. Conversely, when the model did not use data augmentation, adding the Transformer module resulted in effect sizes of − 0.063 and − 0.002 for the BCI IV-2a and BCI IV-2b datasets, respectively. These negative or near-zero effect sizes indicate that the Transformer module alone does not improve, and may even slightly detract from, model performance without data augmentation. These findings also indicate that combining CNN with a Transformer, especially without data augmentation, leads to a decrease in recognition accuracy. This decline is likely due to the introduction of the Transformer module, which increases the model's trainable parameters more than fourfold, thereby exacerbating issues of overfitting. Transformers are equipped with a large number of parameters and layers that are advantageous for capturing complex patterns in extensive datasets but can lead to overfitting when the training data is scarce. Under these conditions, the model may start memorizing noise and specific details of the training set instead of generalizing from it. Lacking sufficient data, the Transformer’s advanced mechanisms, such as multi-head attention, are not fully leveraged. This scenario results in a model that is overly complex for the available data volume, consequently underperforming. These findings align with the results from Keutayeva's research^55,56^.

For the BCI IV-2a and IV-2b datasets, when the Transformer module was removed, using data augmentation resulted in effect sizes of 0.441 and -0.049 for the BCI IV-2a and BCI IV-2b datasets, respectively. This shows that data augmentation alone can have a substantial positive effect in the BCI IV-2a dataset but may have a slightly negative impact in the BCI IV-2b dataset without the Transformer. When the Transformer module was used, adding data augmentation resulted in effect sizes of 0.758 and 0.143, respectively. These results underscore the importance of the Transformer module in improving the model's performance. Its ability to capture global dependencies in the data, especially when combined with data augmentation, significantly boosts the model's effectiveness, particularly in datasets where data augmentation alone may not be sufficient.

For the BCI IV-2a and IV-2b datasets, when the model simultaneously uses both the Transformer and data augmentation, the effect sizes are 0.595 and 0.139, respectively. These results indicate that the combined use of the Transformer and data augmentation significantly enhances model performance on the BCI IV-2a dataset, while the effect is more modest on the BCI IV-2b dataset.

Overall, these results underscore the importance of the Transformer module in enhancing model performance. While data augmentation provides substantial benefits, the Transformer's advanced mechanisms, such as self-attention, are essential for fully leveraging the enriched data environment and capturing complex temporal dependencies in EEG signals. The Transformer's effectiveness is particularly pronounced when combined with data augmentation, as it significantly boosts the model's ability to generalize from enriched data.

### Discussion on the impact of hyperparameters on model performance

We investigated three critical hyperparameters of the CTNet model: token size, the number of heads in the MHA, and the depth of the Transformer module. The CTNet model is sensitive to the settings of these parameters. Our findings suggest that smaller tokens effectively reduce local noise, which facilitates the learning of global features. When decoding EEG signals, capturing the spatial distribution of brainwaves is crucial. Each "head" in a Transformer can be viewed as an independent feature detector, focusing on different dimensions of information. CTNet performs best with a two-head attention mechanism. Two heads represent an optimal balance, sufficient to capture the essential spectral characteristics of $\upmu$ rhythm (8-13Hz) and $\upbeta$ $\text{rhythm}$ (13-30Hz), while a higher number of heads could exceed the processing needs required for the complexity of MI-EEG signals, potentially reducing overall efficiency and effectiveness. Furthermore, CTNet achieves optimal recognition performance with a Transformer encoder of depth 6, mirroring findings from Conformer studies. Feature visualization further affirmed that the Transformer encoder facilitates learning more discriminative features than those extracted without the Transformer.

### Limitations and future work

Although CTNet has demonstrated superior performance in both subject-specific and cross-subject MI-EEG decoding across two datasets, outperforming several advanced methods in terms of recognition accuracy, it still faces certain limitations. Firstly, there is significant room for improvement in CTNet’s recognition accuracy, especially in cross-subject MI-EEG decoding tasks. Secondly, CTNet appears sensitive to specific hyperparameters such as token size, the number of heads in the MHA, and the depth of the Transformer module. This sensitivity might necessitate extensive hyperparameter tuning to achieve optimal performance, which can be time-consuming and computationally demanding. Additionally, the S&R data augmentation strategy does not significantly contribute to the recognition accuracy of subject-specific MI-EEG decoding on the BCI IV-2b dataset. Moving forward, we plan to explore regularization strategies specifically aimed at addressing cross-subject variability, which may enhance the model’s recognition performance in cross-subject MI-EEG decoding. To address the issue of hyperparameter sensitivity, we will attempt to automate the search for optimal hyperparameter combinations using reinforcement learning-based methods. Furthermore, we also intend to explore the use of generative adversarial networks (GANs) to enhance the training dataset.

## Conclusions

In this study, we introduced CTNet, a novel architecture tailored for EEG-based MI classification. Leveraging the strengths of CNN for local feature extraction from EEG signals, CTNet further capitalizes on a Transformer architecture, employing the MHA mechanism to capture the global correlations among these features. Extensive comparative analyses conducted on the BCI IV-2a and IV-2b datasets underscore CTNet's efficacy. Compared to several state-of-the-art methods, CTNet consistently holds a leading position in both subject-specific and cross-subject MI-EEG decoding evaluations. In the subject-specific assessments, CTNet achieved impressive average accuracies of 82.52% on the BCI IV-2a dataset and 88.49% on the BCI IV-2b dataset, with corresponding Kappa values of 0.7670 and 0.7697. In the cross-subject evaluations, CTNet reached average accuracies of 58.64% on the BCI IV-2a dataset and 76.27% on the BCI IV-2b dataset, with Kappa values of 0.4486 and 0.5252, respectively. These results not only demonstrate CTNet's superior performance across both subject-specific and cross-subject settings but also highlight its potential to set new benchmarks in the field.

## Data Availability

The datasets generated and/or analysed during the current study are available in the BCI Competition IV repository. The BCI IV-2a dataset is available for download at the following link: [https://www.bbci.de/competition/download/competition_iv/BCICIV_2a_gdf.zip]. The BCI IV-2b dataset is available for download at the following link: [https://www.bbci.de/competition/download/competition_iv/BCICIV_2b_gdf.zip].
